# Enhancing endurance performance predictions: the role of movement velocity in metabolic simulations demonstrated by cycling cadence

**DOI:** 10.1007/s00421-024-05663-4

**Published:** 2025-02-04

**Authors:** Anna Katharina Dunst, Clemens Hesse, Olaf Ueberschär

**Affiliations:** 1 Institute for Applied Training Science, Department of Endurance Sports, Leipzig, Germany; 2 German Cycling Federation, Frankfurt am Main, Germany; 3 Magdeburg-Stendal University of Applied Sciences, Department of Engineering and Industrial Design, Magdeburg, Germany; 4 Institute for Applied Training Science, Department of Biomechanics, Leipzig, Germany

**Keywords:** Mader’s metabolic simulation, Cycling, Oxygen uptake, Maximal lactate Accumulation rate, Power–velocity profile

## Abstract

**Background:**

Mader’s mathematical model, widely employed for endurance performance prediction, aims to accurately represent metabolic response to exercise. However, it traditionally overlooks dynamic changes in metabolic processes at varying movement velocities.

**Methods:**

This narrative review examined the effect of cycling cadence on its key input parameters, including oxygen demand per Watt ($\text{CE}_{{\dot{\text{V}}}\text{O2}}$), resting oxygen uptake (${{\dot{\text{V}}}\text{O}_{\text{2Base}}}$), maximal oxygen uptake (${{\dot{\text{V}}}\text{O}_{\text{2max}}}$), and maximal blood lactate accumulation rate (*v*La_max_). These findings were integrated into the model to assess cadence-induced variations in predicted power output at maximal aerobic power (MAP), maximal lactate steady state (MLSS), and peak fat oxidation (FAT_max_).

**Results:**

A U-shaped relationship was found between cadence and both $\text{CE}_{{\dot{\text{V}}}\text{O2}}$ and ${{\dot{\text{V}}}\text{O}_{\text{2Base}}}$, while ${{\dot{\text{V}}}\text{O}_{\text{2max}}}$ remained largely cadence-independent within typical cadences. *v*La_max_ exhibited a polynomial increase with cadence, attributed to changes in lactate elimination, suggesting cadence-independent maximal glycolytic flux. Incorporating these findings into Mader’s model considering various scenarios revealed significant cadence-induced variations, with power output differences of up to > 100 W. Using cadence-dependent $\text{CE}_{{\dot{\text{V}}}\text{O2}}$ and ${{\dot{\text{V}}}\text{O}_{\text{2Base}}}$ while maintaining constant ${{\dot{\text{V}}}\text{O}_{\text{2max}}}$ and *v*La_max_ yielded polynomial power output-cadence relationships, with optimal cadences of 84 rpm at MAP, 80 rpm at MLSS, and 70 rpm at FAT_max_. Incorporating cadence-dependent *v*La_max_ produced implausible results, supporting cadence-independent maximal glycolytic flux. A hypothesized cadence-dependent ${{\dot{\text{V}}}\text{O}_{\text{2max}}}$ improved alignment between model predictions and empirical data.

**Conclusion:**

Neglecting dynamic changes in metabolic processes across different movement velocities can lead to inaccurate modelling results. Incorporating cadence alongside established parameters enhances the precision of Mader’s metabolic model for cycling performance prediction.

**Graphical abstract:**

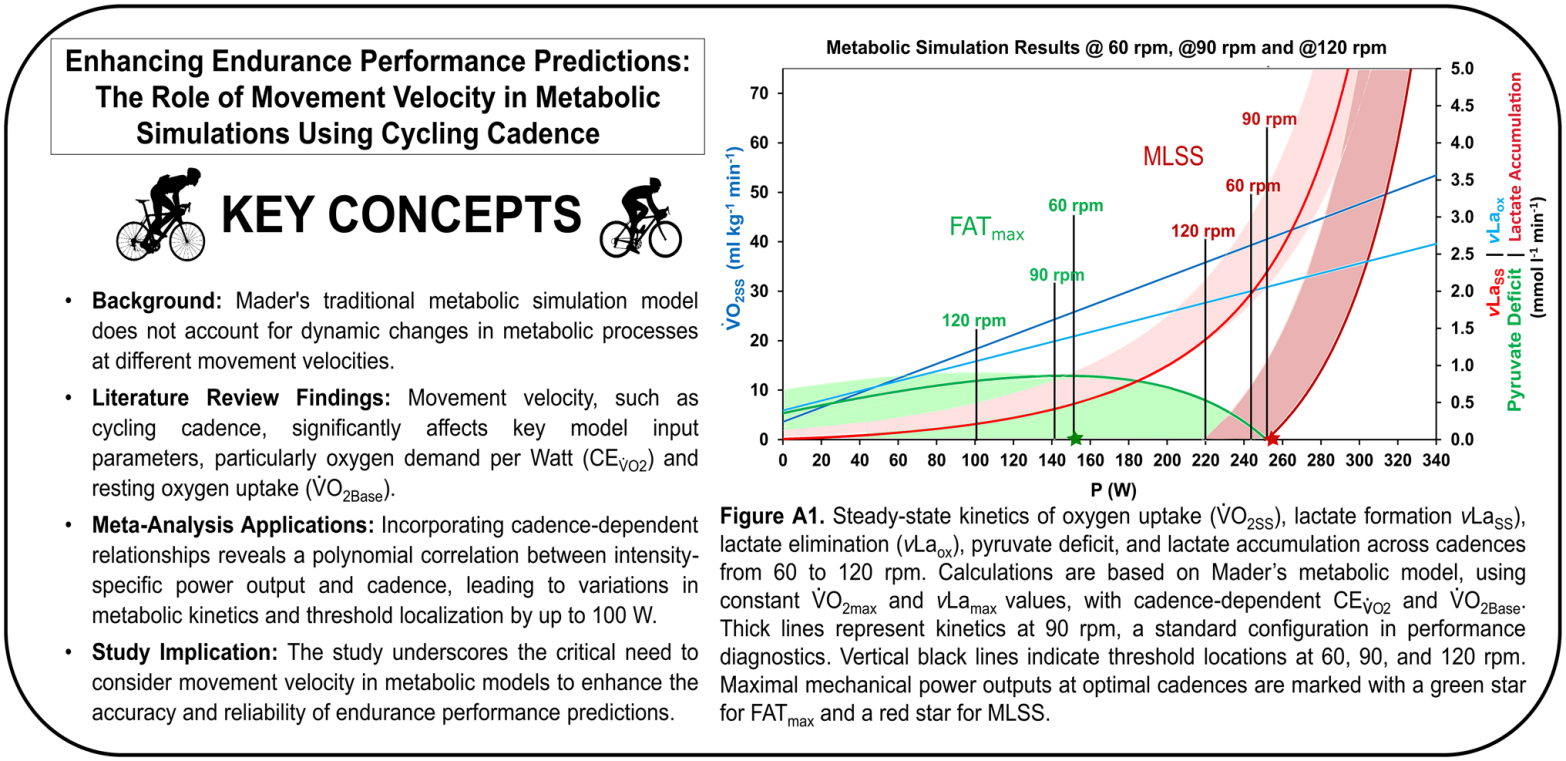

## Introduction

Physiological performance indicators, such as maximal oxygen uptake (${{\dot{\text{V}}}\text{O}_{\text{2max}}}$) and maximal lactate accumulation rate (*v*La_max_), are commonly used to evaluate an athlete;s current physiological performance level (Mavroudi et al. 2023). ${{\dot{\text{V}}}\text{O}_{\text{2max}}}$, a well-established measure of aerobic capacity, represents the highest rate of oxygen consumption attainable during exhaustive exercise and is a primary determinant of endurance performance (Howley et al. 1995). Similarly, *v*La_max_ evaluates anaerobic performance by reflecting the highest rate at which lactate accumulates in the blood during short maximal exercise, serving as a surrogate for exercise-specific maximal glycolytic flux (Mader 1984; Mader and Heck 1986, 1994). Metabolic thresholds, which mark significant metabolic changes across different exercise intensities up to maximal aerobic power (MAP), are sensitive markers of endurance performance, used to delineate intensity zones (Seiler and Tønnessen 2009). The determination of these thresholds using various parameters and methods is a subject of intense debate within the scientific community (Heck and Wackerhage 2024).

Mader's metabolic model, widely used for predicting endurance performance and guiding training, offers a computational alternative to direct assessments of metabolic thresholds by simulating metabolic responses to exercise. Developed by Mader (1984) and refined by Mader and Heck (1986), this model provides a quantitative framework for examining the interplay between oxidative and glycolytic energy systems, expressing oxidative phosphorylation and glycolysis as functions of cytosolic phosphorylation state and muscle power output (Mader 1984, 2003; Mader and Heck 1986). To calculate workload-specific metabolic response, the model requires four key input parameters: Maximal oxygen uptake and maximal lactate accumulation rate define the exercise-specific peak fluxes of oxidative phosphorylation and glycolysis, respectively. The oxygen demand per unit of mechanical power output ($\text{CE}_{{\dot{\text{V}}}\text{O2}}$) and resting oxygen uptake (${{\dot{\text{V}}}\text{O}_{\text{2Base}}}$), derived from the slope and intercept of the linear ${{\dot{\text{V}}}\text{O}_{\text{2}}}$-power output relationship, determine the workload-specific steady-state oxygen uptake (${{\dot{\text{V}}}\text{O}_{\text{2ss}}}$). Normalizing ${{\dot{\text{V}}}\text{O}_{\text{2ss}}}$ by ${{\dot{\text{V}}}\text{O}_{\text{2max}}}$ provides an estimate of muscular energy demand, which, combined with *v*La_max_, allows for the quantification of glycolytic activity. This approach calculates exercise- and workload-specific oxidative phosphorylation and glycolysis activation, particularly under steady-state conditions, enabling the determination of metabolic thresholds, such as maximal lactate steady state (MLSS) and their corresponding exercise intensities.

With the increasing popularity and commercial availability of mobile spiroergometric systems, blood lactate concentration measurement devices, and powermeters, the validity of metabolic models has become a focus of scientific evaluation. While some researchers have found good or acceptable correlations between simulation results and field or laboratory data, particularly between simulated and empirically determined MLSS (Hauser et al. 2014; Ji et al. 2021; Podlogar et al. 2022; Poffé et al. 2024), others have reported notable discrepancies, raising concerns about the practical applicability of the model in performance diagnostics (Poole et al. 2021; Nolte et al. 2022). Some of these discrepancies may be attributed to treating critical input parameters as fixed values or individualized constants. Specifically, the oxygen demand per unit of power output and resting oxygen uptake are often considered fixed parameters. However, research indicates that these critical input parameters are influenced by various factors, including environmental conditions, warm-up protocols, fatigue levels, dietary intake, and movement velocity (Anderson et al. 2006; Dunst et al. 2024; Haase et al. 2024; Pohl et al. 2024). This suggests that they may be dynamic functions of movement velocity with condition-specific variations. Notably, this dynamic aspect is not yet incorporated into standard assessment protocols. In this article, we study the influence of cycling cadence on these critical input parameters and demonstrate the consequent impact on the widely used model of exercise metabolism.

## Methods

A narrative review was conducted to examine the influence of cycling cadence on the following key input parameters for Mader's metabolic model: $\text{CE}_{{\dot{\text{V}}}\text{O2}}$, ${{\dot{\text{V}}}\text{O}_{\text{2Base}}}$, ${{\dot{\text{V}}}\text{O}_{\text{2max}}}$, and *v*La_max_. The literature search utilized PubMed, Google Scholar, Web of Science and Semantic Scholar databases, focusing on peer-reviewed studies investigating the relationship between movement velocity and these parameters. To ensure a thorough historical perspective, university library archives were also consulted, allowing access to early scientific literature on the subject. The extracted data were analyzed to identify patterns and variations across different cadences. Cadence-dependent variations were then integrated into Mader's model, with model algorithms modified to reflect the observed dynamic changes. Simulations using both the traditional and the adjusted model were executed to quantify the effects of cadence-induced parameter changes on predicted power output at metabolic thresholds: MAP, MLSS, and peak fat oxidation (FAT_max_). The review and simulation analysis findings were synthesized to provide a comprehensive understanding of the impact of cycling cadence on metabolic processes and to assess the necessity of incorporating movement velocity into metabolic modelling.

## Review results

### Oxygen uptake and pedaling rate

Previous studies have consistently shown that pedaling rate influences respiratory responses during endurance cycling (Michaelis and Müller 1943; Hess et al. 1963; Israel et al. 1967; Hagberg et al. 1981; Schürch et al. 1976; Böning et al. 1984; Buchanan and Weltman 1985; Chavarren and Calbet 1999; Hintzy et al. 1999; Woolford et al. 1999; Zoldaz et al. 2000; Dunst et al. 2024). During incremental tests, steady-state oxygen uptake increases linearly with workload:

1 $${\dot {\text{V}}}{\text{O}}_2 \left( {\text{P}} \right) = {\text{a}} \cdot {\text{P}} + {\text{b}},$$

This relationship is specific to pedaling rate and converges at maximum aerobic power (Israel et al. 1967; Hughes et al. 1982; Coast and Welch 1985; Chavarren and Calbet 1999; Woolford et al. 1999; Zoldaz et al. 2000). Consistent with previous findings, a recent study showed that ${\dot {\text{V}}}{\text{O}}_{2}$ during incremental exercise up to MAP was the lowest at 60 rpm and the highest at 120 rpm, when comparing pedaling rates of 60 rpm, 90 rpm, and 120 rpm (Dunst et al. 2024). The effect of cadence on the slope (a) and the y-intercept (b) of the linear ${\dot {\text{V}}}{\text{O}}_{2}$–P relationship is opposed. While the slopes of ${\dot {\text{V}}}{\text{O}}_{2}$(P) were the highest at 60 rpm and decreased with cadence, the intercepts were the lowest at 60 rpm and increased significantly with pedaling rate (ibid.). Physiologically, the slope represents the oxygen demand per unit of power output (a = $\text{CE}_{{\dot{\text{V}}}\text{O2}}$), while the intercepts indicate the baseline or resting levels of ${\dot {\text{V}}}{\text{O}}_{2}$ (b = ${{\dot {\text{V}}}{\text{O}}_{\text{2Base}}}$).

**Fig. 1:**
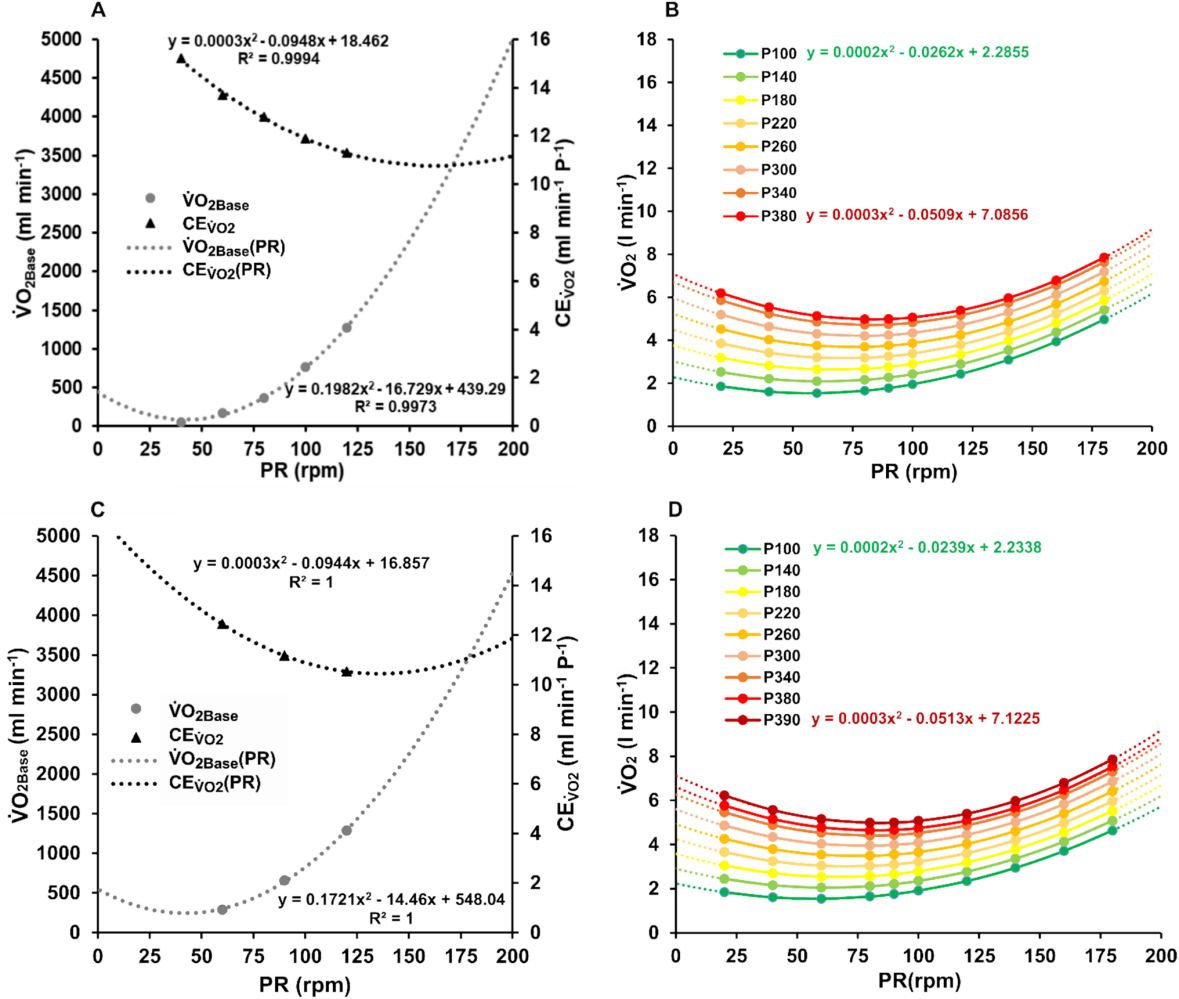
The slope ($\text{CE}_{{\dot{\text{V}}}\text{O2}}$) and the y-axis (${\dot {\text{V}}}{\text{O}}_{\text{2Base}}$) of the linear ${\dot {\text{V}}}{\text{O}}_{2}$–P relationship as a function of pedaling rate. The data used to obtain these models were published by (**A**) Coast and Welch (1985) and (**C**) Dunst et al. (2024). Based on each dataset, a U-shaped ${\dot {\text{V}}}{\text{O}}_{2}$–PR relationship was derived from each workload ranging from 100 to 380 W and 390 W, respectively. The cadences corresponding to the function minimum increased from 60 to 85 rpm in both cases (**C**, **D**)

A reanalysis of previously published data suggests that both the slope and the y-intercept of the linear ${\dot {\text{V}}}{\text{O}}_{2}$–P relationship change systematically with pedaling rate in a quadratic manner (Israel et al. 1967; Gaesser and Brooks 1975; Seabury et al. 1977; Hagberg, 1981; Coast and Welch 1985; Francescato et al. 1995; Chavarren and Calbet 1999; Woolford et al. 1999; Dunst et al. 2024). A representative example of this pattern is shown in Fig. 1A. To validate the assumed pattern, ${\dot {\text{V}}}{\text{O}}_{2}$–P relationships were interpolated for pedaling rates ranging from 20 to 180 rpm, and the workload-specific oxygen uptake-pedaling rate function (${\dot {\text{V}}}{\text{O}}_{2}$(PR)) was computed from 100 to 380 W (MAP), as displayed in Fig. 1B. As previously reported, a U-shaped relationship of the oxygen-uptake-pedaling rate should appear for each intensity, with an increasing and right-shifting extremum up to MAP (Banister and Jackson 1967; Israel et al. 1967; Seabury et al. 1977; Hagberg et al. 1981; Böning et al. 1984; Buchanan and Weltman 1985; Coast and Welch 1985; Chavarren et al., 1999; Hintzy et al. 1999; Woolford et al. 1999; Argentin et al. 2006). The identified model functions were applied to the recently published data by Dunst et al. (2024) and checked for validity. The results are illustrated in Fig. 1C and D.

This analysis provides strong evidence for a systematic relationship between $\text{CE}_{{\dot{\text{V}}}\text{O2}}$ and ${\dot {\text{V}}}{\text{O}}_{\text{2Base}}$ with cadence. The identified model functions effectively capture this relationship, as evidenced by the physiologically plausible and empirically observable changes in the workload-specific ${\dot {\text{V}}}{\text{O}}_{2}$–cadence relationship with increasing intensity. The cadence-dependent relationships observed for $\text{CE}_{{\dot{\text{V}}}\text{O2}}$ and ${{\dot{\text{V}}}\text{O}_{\text{2Base}}}$ highlight the need to account for the influence of movement velocity when modelling metabolic responses during exercise.

In contrast to the cadence-dependent relationships observed for $\text{CE}_{{\dot{\text{V}}}\text{O2}}$ and ${{\dot {\text{V}}}{\text{O}}_{\text{2Base}}}$, maximal oxygen uptake appears largely independent of pedaling rate, as no clear systematic pattern emerges from previous studies (Michaelis and Müller 1943; McKay and Banister 1976; Schürch et al. 1976; Pivarnik et al. 1988; Buchanan and Weltman 1985; Moore and Tong 1994; Woolford et al. 1999; Brickson et al. 2022). Evidence supporting this independence includes mostly non-significant differences in cadence-specific peak oxygen uptake (Michaelis and Müller 1943; Schürch et al. 1976; Pivarnik et al. 1988; Moore and Tong 1994; Woolford et al. 1999; Zoldaz et al. 2000; Brickson et al. 2022) and inconsistent findings across studies reporting significant differences (McKay and Banister 1976; Buchanan and Weltman 1985). For instance, McKay and Banister (1976) observed the lowest peak oxygen uptake at 60 rpm during an incremental test at 60, 80, 100, and 120 rpm, while Buchanan and Weltman (1985) found the highest ${{\dot {\text{V}}}{\text{O}}_{\text{2peak}}}$ at 60 rpm when comparing cadences of 60, 90, and 120 rpm. These contradictory results suggest that test conditions may have influenced outcomes rather than reflecting true physiological phenomena. Supporting this hypothesis, McKay and Banister (1976) reported significantly reduced maximal heart rates despite extended time to exhaustion at 60 rpm, indicating that muscular fatigue likely precedes maximum central circulation stress. Additionally, their treadmill tests revealed no significant differences in peak ${\dot {\text{V}}}{\text{O}}_{2}$ at varying running speeds or inclines, aligning with results reported by Silva et al. (2021). These observations suggest a lack of compelling physiological rationale for a movement velocity-dependent relationship. The independence of ${{\dot {\text{V}}{\textrm{O}}}_{\textrm{2max}}}$ from cadence is further corroborated by the convergence of linear cadence-specific $\text{CE}_{{\dot{\text{V}}}\text{O2}}$–P function curves at MAP, as demonstrated in multiple studies (Israel et al. 1967; Hughes et al. 1982; Coast and Welch 1985; Chavarren and Calbet 1999; Woolford et al. 1999; Zoladz et al. 2000; Dunst et al. 2024). Collectively, these findings suggest that ${{\dot {\textrm{V}}{\text{O}}}_{\textrm{2max}}}$ is a relatively stable physiological parameter, largely unaffected by movement velocity across the investigated cadence ranges. This independence is crucial for reliable metabolic modelling across various cadences. However, conflicting results indicate that a cadence dependence cannot be entirely ruled out, necessitating further research to definitively clarify the relationship between ${{\dot {\textrm{V}}{\text{O}}}_{\textrm{2max}}}$ and movement velocity.

### Blood lactate accumulation rate and pedaling rate

The measurement of changes in blood lactate concentration is crucial in assessing anaerobic performance (Driss and Vandewalle 2013). Maximal anaerobic lactic power can be described by measuring the maximal blood lactate accumulation rate as a surrogate for maximal anaerobic glycolytic energy flux (Mader 1984, 1994, 2003; Heck et al. 2003). *v*La_max_ can be determined by measuring the change in blood lactate concentration (ΔBLC) following short maximal sprints, divided by the exercise duration (T) minus an initial lactate-free period (t_alac_) of approximately 2–3 s (Mader 1984, 1994, 2003; Heck et al. 2003; Adam et al. 2015; Dunst et al. 2023a, b; Langley et al. 2024; Haase et al. 2024):

2 $$v {\text{La}}_{\max } = \frac{{\Delta {\text{BLC}}}}{\text{T} - \text{t}_{\text{alac}}}$$

**Fig. 2:**
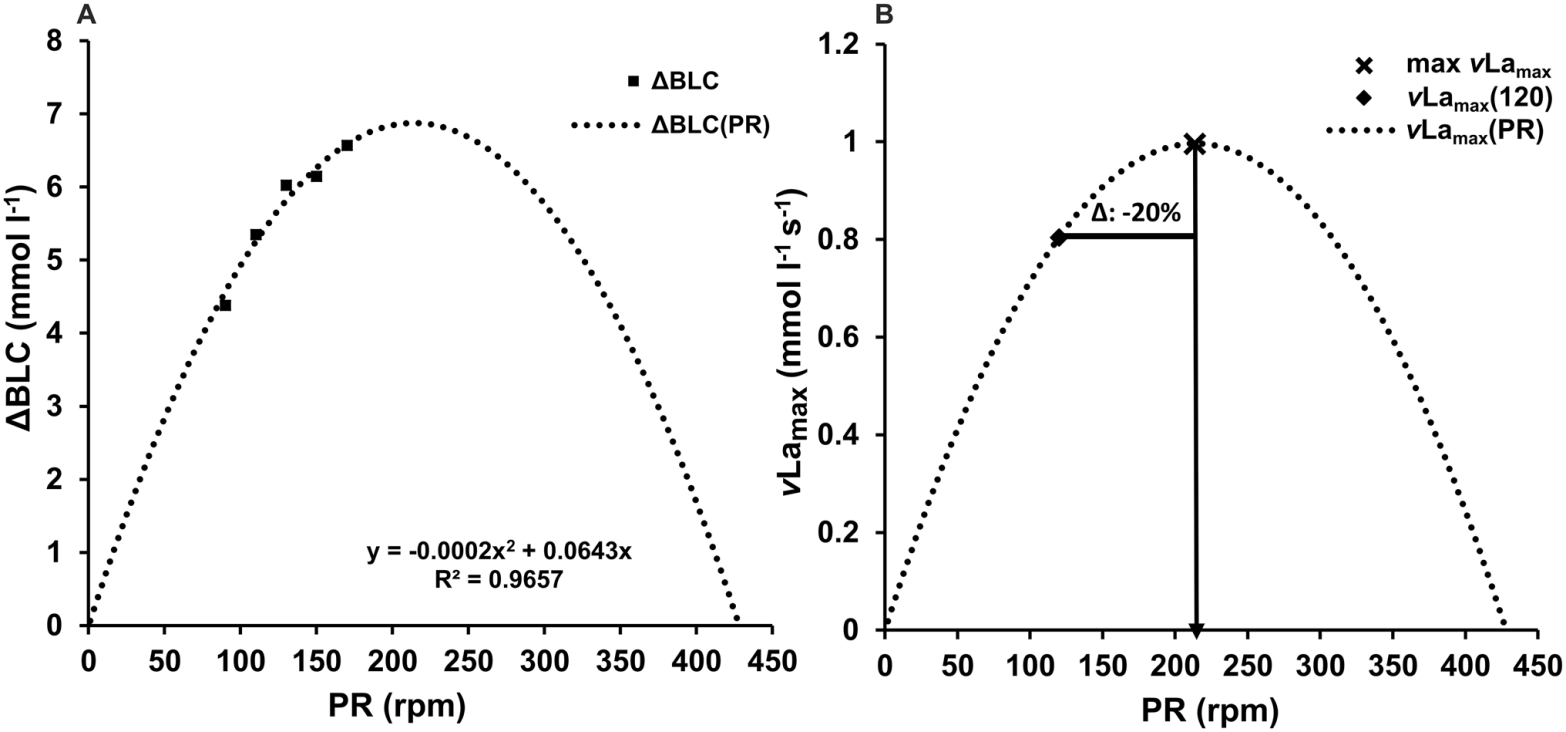
**A** Relationship between pedaling rate and the change in blood lactate concentration (ΔBLC) during 10-s maximal sprints on a cycle ergometer, as reported by Haase et al. (2024). The data show a systematic increase in ΔBLC with increasing pedaling rate, which can be described by a second-order polynomial function. **B** Application of the function to the *v*La_max_ of 0.8 mmol l^−1^ s^−1^ at 120 rpm, as reported by Dunst et al. (2024). This example illustrates a 20% discrepancy between the measured value and the predicted maximum of 1.0 mmol l^−1^ s^−1^ occurring at 214 rpm, highlighting the potential underestimation of *v*La_max_ in standard testing protocols

Recent research by Haase et al. (2024) investigated the influence of cadence on ΔBLC during 10-s maximal cycling sprints. Comparing ΔBLC across pedaling rates of 90, 110, 130, 150, and 170 rpm, the study found that higher cadences result in greater blood lactate accumulation. A closer examination of the data suggests a systematic increase in ΔBLC with increasing cadence, described by a second-order polynomial relationship (see Fig. 2). This pattern was observed for all participants with an average optimal pedaling rate of 236 ± 60 rpm and an *R*^2^ of 0.98 ± 0.02 (unpublished data). Assuming a cadence-independent alactic time span, this finding implies a strong dependence of *v*La_max_ on pedaling rate.

However, the observed polynomial relationship between maximal blood lactate accumulation and cadence may be an artefact of current measurement methodologies. As direct measurement of glycolysis rate is currently limited to complex, localized PMNR spectroscopy, the gold standard remains the maximum blood lactate accumulation rate as a surrogate for maximum global glycolytic flux. However, this rate only measures the difference between lactate formation and elimination after diffusion into the blood. Assuming full muscle mass activation during maximal efforts (Sargeant 1994), actual lactate formation should be exercise-specific, similar to ${\dot{\text{V}}\text{O}}_{2\text{max}}$, but should reach a maximum regardless of movement velocity. The influence of cadence on measurable blood lactate accumulation demonstrated by Haase et al. (2024) could therefore be due to cadence-dependent lactate elimination, influenced by the proportion of oxidative muscles in the active muscle mass (Dunst et al. 2024). This interpretation suggests that the observed cadence-dependent variations in *v*La_max_ may not accurately reflect true changes in maximal glycolytic flux, but rather differences in lactate kinetics.

## Application of the review results to the metabolic model

Based on our findings, the slope and y-intercept of the linear ${\dot{\text{V}}}{\text{O}}_{2}$–P relationship, as well as the maximal blood lactate accumulation rate, can be expressed as functions of cadence. Applying these model functions to Mader’s metabolic model allows us to examine the model's sensitivity to movement velocity. Specifically, we can calculate cadence-specific power outputs at various metabolic thresholds, including the (FAT_max_, synonymous with Mader’s maximum pyruvate deficit) and the maximal lactate steady state (Mader 1984, 2003; Mader and Heck 1986), can be calculated to test the results for cadence effects.

To identify metabolic thresholds using Mader’s approach, knowledge of ${{\dot {\text{V}}}{\text{O}}_{\text{2ss}}}$ and glycolysis (*v*La_ss_) is essential (Mader 1984, 2003; Mader and Heck 1986). Both ${{\dot {\text{V}}}{\text{O}}_{\text{2ss}}}$ and *v*La_ss_ can be expressed using Michaelis–Menten equations that account for enzyme activation based on substrate concentration and performance capacities (ibid.)

The intensity-specific activity of ${{\dot {\text{V}}}{\text{O}}_{\text{2ss}}}$ can be expressed via the following equation knowing the exercise-specific V̇O_2max_ (Mader and Heck 1986; Mader 2003):

3 $${\dot {\text{V}}}{\text{O}}_{{2{\text{ss}}}} = \frac{{{\dot {\text{V}}}{\text{O}}_{{2{\text{max}}}} }}{{1 + \frac{{{\text{ks}}1}}{{\left[ {{\text{ADP}}} \right]^{2} }}}}$$

where [ADP] (mmol·kgm⁻^1^) indicates current energy demand and ks1 = 0.0635 (mmol·kgm⁻^1^)^2^ reflects the 50% activation constant of oxidative phosphorylation.

The intensity-specific activity of* v*La_ss_ can be expressed via the following equation knowing the exercise-specific maximal lactate accumulation rate (Mader and Heck 1986; Mader 2003):

4 $$  v \text{La}_{{{\text{ss}}}} { = }\frac{{v\text{La}_{{{\text{max}}}} }}{{{1 + }\frac{{{\text{ks2}}}}{{{\text{[ADP]}}^{{3}} }}}}$$

where ks2 = (1.1 mmol·kgm⁻^1^)^3^ represents the 50% activation constant of glycolysis.

The unknown [ADP] can be derived from the following equation:

5 $$ \left( {\frac{{{\text{ks1}} \cdot {\dot {\text{V}}}{\text{O}}_{{{\text{2ss}}}} }}{{{\dot {\text{V}}}{\text{O}}_{{{\text{2max}}}} { - }{\dot {\text{V}}}{\text{O}}_{{{\text{2ss}}}} }}} \right)^{{\frac{{1}}{{2}}}} { = }\left[ {{\text{ADP}}} \right]{ = }\left( {\frac{{{\text{ks2}} \cdot v\text{LA}_{{{\text{ss}}}} }}{{v\text{La}_{{{\text{max}}}} { - }v\text{LA}_{{{\text{ss}}}} }}} \right)^{{\frac{{1}}{{3}}}}$$

Lactate elimination primarily occurs in active muscle and is a linear function of oxygen consumption, influenced by the amount of pyruvate/lactate oxidized per unit of oxygen and the distribution volume (Mader and Heck 1986). The rate of lactate oxidation (*v*La_ox_) can be calculated as:

6 $$ {v\text{La}}_\text{ox} = {\text{CE}}_{\text{La} \cdot \text{{O}}_2} \cdot \frac{{\dot{\text{V}}}\text{O}_\text{{ss}}}{{\text{Vol}}_\text{{La}}}$$

In the demonstrated calculations, a lactate-equivalent of CE_La.O2_ = 0.02049 mmolLa (mlO_2_)^−1^ and a lactate distribution volume Vol_La_ = 0.4 were used.

The pyruvate deficit (PD) can be calculated as the difference between the capacity to metabolize pyruvate via oxidative phosphorylation and the pyruvate available due to the current glycolytic flux:

7 $${\text{PD = }}v\text{La}_{{\text{ox}}} { - }v\text{La}_{\text{ss}}$$

The term 'maximum pyruvate deficit' indicates the least saturation of oxidative phosphorylation with pyruvate, corresponding to the intensity with the highest fat combustion, often referred to as FAT_max_:

8 $${\text{FAT}}_{\text{max}}\text{=}{\text{max}}\text{(PD)}$$

The MLSS is defined as the point where lactate formation equals lactate elimination, corresponding to a pyruvate deficit of zero. It can be calculated as:

9 $${\text{MLSS = }}v\text{La}_{\text{ox}} { - }v\text{La}_{\text{ss}} \mathop {\text{=}}\limits^{!} 0$$

Steady-state oxygen uptake can be assessed using spirometry and described via a linear relationship between ${{\dot {\text{V}}}{\text{O}}_{\text{2ss}}}$ and workload:

10 $${\dot{\text{V}}}{\text{O}}_{{{\text{2ss}}}} {\text{ = CE}}_{{{\dot {\text{V}}}{\text{O}}_{{2}} }}\cdot {\text{P + }}{\dot {\text{V}}}{\text{O}}_{{{\text{2Base}}}}$$

Using this equation, the corresponding work rate for any level of ${\dot {\text{V}}}{\text{O}}_{\text{2ss}}$ can be calculated as:

11 $${\text{P = }}\frac{{{\dot {\text{V}}}{\text{O}}_{{{\text{2SS}}}} { - }{\dot {\text{V}}}{\text{O}}_{{{\text{2Base}}}} }}{{{\text{CE}}_{{{\dot {\text{V}}}{\text{O}}_{{2}} }} }}$$

Regarding *v*La_max_ as an input parameter for modelling an athlete’s workload-specific glycolytic activity, it is crucial to determine whether the maximum attainable blood lactate accumulation rate or the maximal value at a specific cadence is required. The observed dependence of ΔBLC and the assumed dependence of *v*La_max_ on cadence can lead to different implications for Mader's metabolic simulation with different outcomes. If the true maximum *v*La_max_ is required, there is a risk of underestimating glycolytic flux at higher cadences than tested, potentially leading to inaccurate assessments of intensity-specific glycolytic activity. Current recommendations typically use a cadence of 120–130 rpm for *v*La_max_ testing (Hauser et al. 2014; Adam et al. 2015; Podlogar et al. 2022; Dunst et al. 2023a, b; Langley et al. 2024; Poffé et al. 2024). Recent findings suggest that maximum *v*La occurs at pedaling rates exceeding 170 rpm (Haase et al. 2024); much higher than previously assumed. Consequently, the intensity guidelines derived from the model may overestimate the athlete's metabolic capabilities. If a cadence-specific* v̇*La_max_ is required, the risk of overestimating or underestimating anaerobic-lactic power depends on the cadence used for diagnostic testing. In this scenario, it is essential to consider cadence-specific glycolytic activity in each calculation step to ensure the accuracy of the model’s predictions.

To investigate the effect of cadence on modelling outcomes, we incorporated the cadence dependencies of the input parameters reported by Dunst et al. (2024) into Mader’s model, considering two different scenarios. In the first scenario, the oxygen demand per unit of power output and resting oxygen uptake were integrated as functions of pedaling rate, while *v*La_max_ was assumed constant at a global maximum of 1.0 mmol l^−1^ s^−1^. This leads to modifications in Eqs. (10 and 11):

12 $${\dot {\text{V}}}{\text{O}}
_{{{\text{2ss}}}} {\text{(PR) = CE}}_{{{\dot {\text{V}}}{\text{O}}_{{2}} }} {\text{(PR)}} \cdot {\text{P(PR) + }}{\dot {\text{V}}}{\text{O}}
_{{{\text{2Base}}}} {\text{(PR)}}$$

13 $${\text{P(PR) = }}\frac{{{\dot {\text{V}}}{\text{O}}
_{{{\text{2SS}}}} {\text{(PR) - }}{\dot {\text{V}}}{\text{O}}_{{{\text{2Base}}}} {\text{(PR)}}}}{{{\text{CE}}_{{\dot {\text{VO}}_{{2}} }} {\text{(PR)}}}}$$

where $${\text{CE}}_{{{\dot {\text{V}}}{\text{O}}_{{2}} }} {\text{(PR) = 0}}{.0004} \cdot {\text{PR}}^{{2}} { - 0}{\text{.094}} \cdot {\text{PR + 16}}{.857}$$ and $${\dot{\text{V}}}{\text{O}}_{{{\text{2Base}}}} {\text{(PR) = 0}}{.172} \cdot {\text{PR}}^{{2}} { - 14}{\text{.460}} \cdot {\text{PR + 548}}{.03}9$$.

In the second scenario, we assumed that $\text{CE}_{{\dot{\text{V}}}\text{O2}}$, ${{\dot {\text{V}}}{\text{O}}_{\text{2Base}}}$, and *v*La_max_ all depend on cadence, leading to the following modification in Eq. (4):

14 $$\mathop {{v}}\!{\text{La}}_{{{\text{ss}}}} {\text{(PR) = }}\frac{{\mathop {{v}}\! {\text{La}}_{{{\text{max}}}} \left( {{\text{PR}}} \right)}}{{{1 + }\frac{{{\text{ks2}}}}{{{\text{[ADP]}}^{{3}} }}}}$$

where $v\text{La}_{{{\text{max}}}} \left( {{\text{PR}}} \right){ = - 0}{\text{.0002}} \cdot {\text{PR}}^{{2}} { + 0}{\text{.00931}} \cdot {\text{PR}}$. The modification implies substitution of *v*La_ss_ in all relevant equations with the function *v*La_ss_(PR).

To compare both approaches, the power output corresponding to a fixed cadence of 90 rpm was calculated using the traditional cadence-independent method. In all scenarios, a ${{\dot {\text{V}}}{\text{O}}_{\text{2max}}}$ of 62.9 ml kg^−1^ min^−1^ was used, according to our recently published data (Dunst et al. 2024).

## Simulation results

The integration of cadence-dependent input parameters into Mader's model revealed significant influences on predicted power outputs at metabolic thresholds. Two scenarios were analyzed: scenario 1, in which a fixed *v*La_max_ was utilized, and scenario 2, in which a cadence-dependent *v*La_max_ was employed.

Both scenarios demonstrated polynomial relationships between cadence and threshold-specific power outputs. In scenario 1, the optimal cadences (PR_opt_) for intensity-specific maximal power output, corresponding to the cadences at the function maxima, were found to be 70 rpm at FAT_max_ and 80 rpm at MLSS. In scenario 2, the PR_opt_ values shifted to 38 rpm at FAT_max_ and 63 rpm at MLSS. Notably, the MAP–PR relationship remained consistent across both scenarios, yielding a PR_opt_ of 84 rpm as the respective calculation is independent of *v*La_max_.

**Fig. 3:**
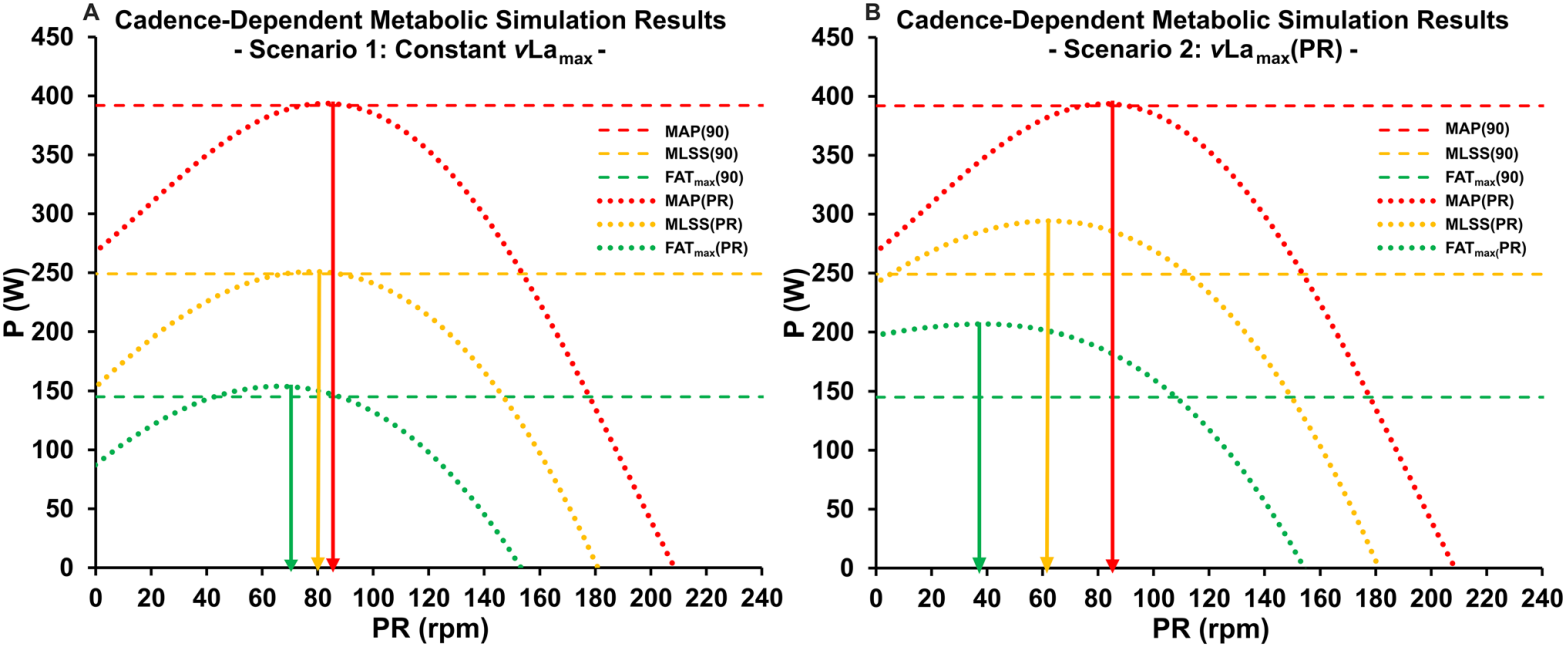
Relationship between pedaling rate and maximum aerobic power (MAP), power output at maximal lactate steady state (MLSS), and power output at peak fat oxidation (FAT_max_), calculated by modelling the slope ($\text{CE}_{{\dot{\text{V}}}\text{O2}}$) and y-intercept (${{\dot {\text{V}}}{\text{O}}_{\text{2Base}}}$) of the ${\dot {\text{V}}}{\text{O}}_{2}$-power output relationship as functions of pedaling rate. Figure (**A**) depicts the results using a fixed maximal blood lactate accumulation rate (*v*La_max_), while figure (B) shows the same relationships with *v*La_max_ modelled as a function of pedaling rate. The modifications to the equations account for the dynamic changes in oxidative phosphorylation and glycolysis across different cadences, resulting in polynomial workload–pedaling rate relationships. For comparison, the dashed horizontal lines represent metabolic threshold power outputs calculated at a fixed cadence of 90 rpm, generalized across all pedaling rates

A comparison of power outputs calculated using the traditional cadence-independent method with those derived from the adapted cadence-dependent approaches revealed notable mean deviations in scenario 1: 108 ± 116 W for MAP, 64 ± 72 W for MLSS, and 35 ± 42 W for FAT_max_. In scenario 2, the mean deviations were slightly higher, with values of 108 ± 116 W for MAP, 65 ± 66 W for MLSS, and 56 ± 31 W for FAT_max_. At a cadence of 90 rpm, the respective power output calculated using the traditional method and scenario 1 were identical across all thresholds. However, in scenario 2, the respective power output at MLSS and FAT_max_ were 15 W higher than those calculated using traditional methods at the same cadence. Figure 3A and B provide a comprehensive visual representation of the simulation outcomes, juxtaposing the results from both cadence-dependent modelling scenarios with the traditional cadence-independent approach.

## Discussion

Incorporating the identified cadence-dependent relationships for $\text{CE}_{{\dot{\text{V}}}\text{O2}}$, ${{\dot {\text{V}}}{\text{O}}_{\text{2Base}}}$, and *v*La_max_ into the metabolic model allows for a detailed exploration of Mader’s metabolic simulation sensitivity to variations in cadence. The primary finding of this analysis is that, contrary to previous assumptions and current methodological recommendations, the results of the metabolic simulation are indeed dependent on movement velocity, in this case the pedaling rate. This conclusion is supported by various empirical studies demonstrating a polynomial relationship between intensity-specific power output and cadence, wherein the optimal cadence, defined as the pedaling rate that maximizes mechanical power output at a given intensity, systematically increases with rising exercise intensity (Coast and Welch 1985; Zoladz et al. 2000; Foss and Hallén, 2004; Dunst et al. 2024). The ability to replicate these empirically observed patterns by incorporating cadence-dependent relationships enhances the validity of the presented approach. Especially, when *v*La_max_ was held constant, the modelled cadence–power output relationships exhibited optimal pedaling rates at the assessed metabolic thresholds (FAT_max_, MLSS, MAP) that aligned closely with recently published data (Dunst et al. 2024). In scenario 1, the optimal cadence increased from 70 rpm at FAT_max_ to 80 rpm at MLSS and 84 rpm at MAP. The minor discrepancies between empirical observations and the simulation results may be attributed to the higher *v*La_max_ value used in the current modelling approach, which shifts the threshold-specific polynomial relationships downward.

Incorporating *v*La_max_ as a function of cadence in scenario 2 resulted in significantly lower optimal cadences for FAT_max_ (38 rpm) and MLSS (63 rpm). This discrepancy can be attributed to reduced maximal blood lactate accumulation at lower pedaling rates, which diminishes the intensity-specific activation of glycolysis and shifts the localization of metabolic thresholds upward. These findings contrast with empirical observations in the literature, which generally reports optimal cadences for endurance performance in the range of 60–90 rpm, depending on exercise intensity (Böning et al. 1984; Coast and Welch 1985; Woolford et al. 1999; Chavarren and Calbet 1999; Zoladz et al. 2000; Foss and Hallén, 2004; Argentin et al. 2006; Dunst et al. 2024). Furthermore, comparing the modelling results and the traditional approach at a fixed cadence of 90 rpm reveals a difference up to 34 W in scenario 2. This substantial discrepancy is in stark contrast to the highly predictive accuracy of the MLSS at a fixed cadence, as consistently shown in various studies (Hauser et al. 2014; Ji et al. 2021; Podlogar et al. 2022; Poffé et al. 2024).

When compared to the recently published findings of our group, significant differences can be observed, particularly at lower pedaling rates. The metabolic simulation employed in the current analysis predicts significantly higher power outputs at lower pedaling rates compared to the empirical relationships reported by Dunst et al. (2024). This discrepancy may be due to several factors, including the limited number of data points in the previous study, which could have affected the accuracy and robustness of the derived model. Additionally, the assumption of a second-order polynomial function, along with the inclusion of an artificial grid point at zero movement velocity, may have led to an underestimation of performance at lower pedaling rates. This assumption aligns with fatigue-free power-velocity relationships but may not accurately reflect dynamics at submaximal intensities and lower cadences.

**Fig. 4:**
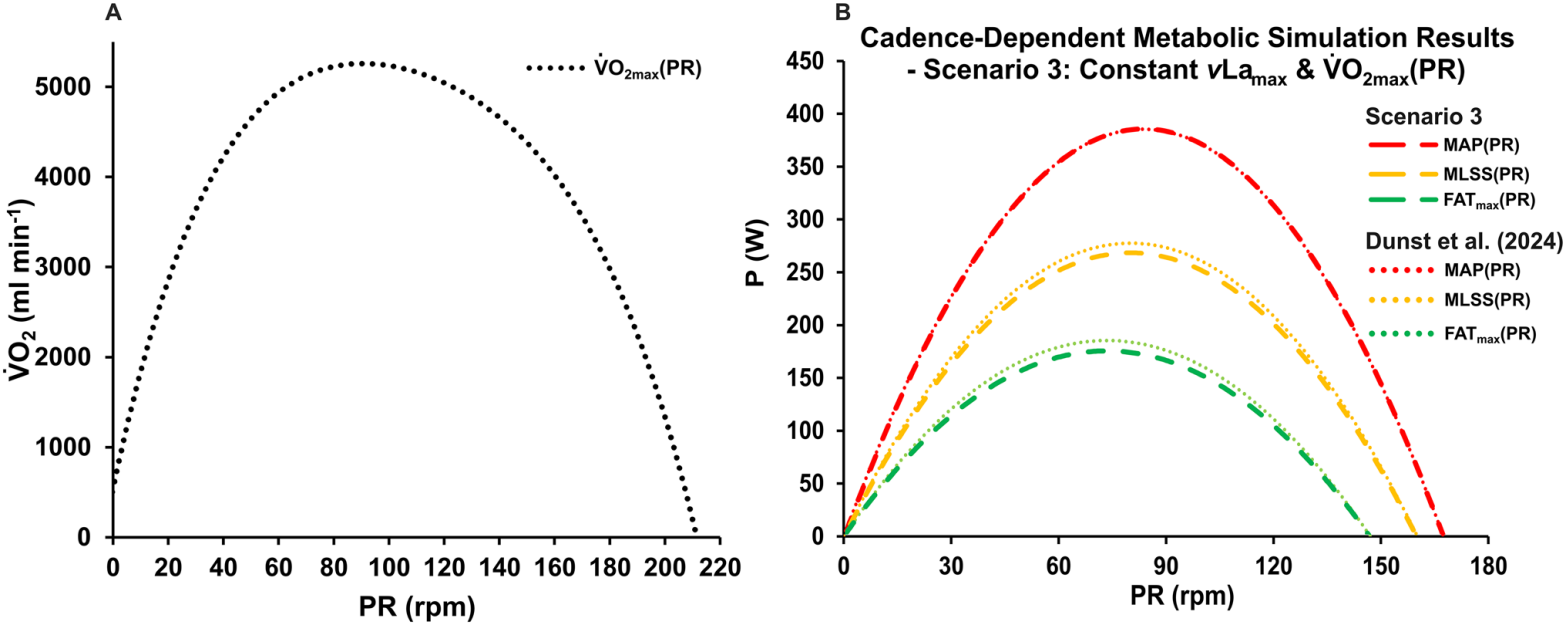
Mathematically reconstructed relationship between ${\dot{\text{V}}\text{O}}_{2\text{max}}$ and cadence (**A**) and predicted power output at MAP, MLSS and FAT_max_ incorporating cadence-dependent functions of $\text{CE}_{{\dot{\text{V}}}\text{O2}}$, ${{\dot {\text{V}}}{\text{O}}_{\text{2Base}}}$ and ${\dot{\text{V}}\text{O}}_{2\text{max}}$ into Mader’s metabolic model (**B**). Calculations were conducted with a constant maximal *v*La_max_ of 0.8 mmol l^−1^ s^−1^, as reported by Dunst et al. (2024). Dotted lines represent results from Dunst et al. (2024), while dashed lines indicate the current modelling results

Alternatively, the discrepancy between empirical findings and model predictions may also be explained by yet unidentified systematic changes in maximal oxygen uptake with movement velocity. Although current research has not established a clear pattern in the ${\dot{\text{V}}\text{O}}_{2\text{max}}$–cadence relationship, the existence of such an association cannot be entirely ruled out. To explore this possibility, we attempted to mathematically reconstruct our recently published empirical findings on the MAP–PR relationship using Eq. (11). This reconstruction yielded the ${\dot{\text{V}}\text{O}}_{2\text{max}}$–PR relationship illustrated in Fig. 4. When incorporated into the metabolic model (scenario 3) via an adjustment to Eq. (3), the simulation outcomes closely aligned with those reported by Dunst et al. (2024), suggesting that including a cadence-dependent ${\dot{\text{V}}\text{O}}_{2\text{max}}$ could potentially reconcile model predictions with empirical observations.

Furthermore, Fig. 4 indicates that previous studies investigating the ${\dot{\text{V}}\text{O}}_{2\text{max}}$–movement velocity relationship may have failed to detect a systematic pattern due to the relatively minor dependency observed within typical testing cadences, which likely falls within the reported margin of error (up to 5%) for ${\dot{\text{V}}\text{O}}_{2\text{max}}$ determination via spiroergometry (Vickers 2003). Further research is needed to test this hypothesis and reconcile the modelling approach with empirical observations. This should include the collection of more comprehensive datasets across a wider range of cadences and the exploration of alternative functional forms that may better capture the underlying physiological processes.

Finding an impact of cadence on metabolic simulations requires a critical reflection of the current methodology used to generate metabolic profiles based on Mader’s model. While accurately determining ${\dot{\text{V}}\text{O}}_{2\text{max}}$ and *v*La_max_ is of significant scientific importance (Beneke et al. 2007; Hauser et al. 2011; Nitzsche et al. 2017; Dunst et al. 2023a,b; Haase et al. 2024; Langley 2024), the dependence of the ${\dot{\text{V}}\text{O}}_{2\text{max}}$–P relationship on cadence has not been adequately addressed. Consequently, metabolic thresholds and derived intensity recommendations are often calculated in a generalized manner (as shown in Fig. 3A and B), suggesting that current modelling approaches may not accurately reflect physiological reality. Recent studies have demonstrated that power output at specific metabolic states is influenced by pedaling rate (Zoladz et al. 2000; Argentin et al. 2006; Leo et al. 2022; Dunst et al. 2024).

Regarding *v*La_max_ determination, our results suggest that the actual maximum value should be used as the input parameter for Mader’s model, neglecting a possible dependence on pedaling rate. While incorporating the *v*La_max_–PR relationship into the model with reconstructed cadence-dependent ${\dot{\text{V}}\text{O}}_{2\text{max}}$ also improves alignment with empirical curves, it still leads to significant overestimation at low cadences and underestimation at high cadences. This finding supports the hypothesis that the observed cadence dependence of ΔBLC is more a methodological artefact than a physiologically valid dependence of maximum glycolytic flux on movement velocity. However, the relationship between ΔBLC and cadence identified by Haase et al. (2024) should not be overlooked. The application of a *v*La_max_ value corresponding to a lower cadence (e.g., 120–130 rpm) as an input parameter may result in an overestimation of intensity-specific power output at various metabolic thresholds in comparison to the true maximum occurring at higher cadences (> 170 rpm). To illustrate, applying a calculated cadence-specific *v*La_max_ of 0.8 mmol l^−1^ s^−1^ at 120 rpm could result in an overestimation of 19 W at both FAT_max_ and MLSS compared to the maximum of 1.0 mmol l^−1^ s^−1^ at 214 rpm.

These findings highlight the need for a more sophisticated approach to metabolic simulation models that accounts for the influence of movement velocity, as exemplified by pedaling rate. Neglecting this factor could result in erroneous predictions of power output and metabolic responses, potentially leading to flawed performance assessments and suboptimal training prescriptions. To address this limitation, future research should focus on developing cadence-specific metabolic simulation models or incorporating cadence as a variable within existing models. By refining metabolic simulation models to account for movement velocity and other relevant factors, researchers and practitioners can enhance the accuracy and reliability of endurance performance predictions, ultimately enabling more personalized and effective training programs and competition strategies for athletes across various sports and disciplines.

### Practical applications

The findings of this study have significant implications for the application of Mader’s metabolic simulation model and its derived products in exercise physiology and sports science. Researchers and practitioners must critically evaluate the nature of input parameters, particularly *v*La_max_ and ${\dot{\text{V}}\text{O}}_{2\text{max}}$, to determine whether they represent exercise-specific absolute maxima, cadence-specific values, or potentially inadequate correlations with specific exercise types. This distinction is crucial as it substantially influences the model's precision and applicability different athletic populations and exercise modalities.

For athletes whose performance is highly dependent on movement velocity, the integration of movement velocity effects on $\text{CE}_{{\dot{\text{V}}}\text{O2}}$ and ${{\dot {\text{V}}}{\text{O}}_{\text{2Base}}}$ into the model is recommended. This refinement improves the accuracy of performance predictions by accounting for the metabolic cost variations associated with different velocities. In contrast, for athletes who primarily train and compete under conditions with minimal velocity fluctuations, such as on flat terrain or at constant speeds, the model's sensitivity to velocity changes may be less critical. However, even in these cases, establishing the ${{\dot {\text{V}}}{\text{O}}_{\text{2}}}$–power relationship at a constant, sport-specific movement velocity is essential for precise predictions.

For all athletes, dynamic changes in *v*La_max_ with movement velocity likely should not be incorporated into the model. Instead, emphasis should be placed on accurately determining the true exercise-specific maximum values. Recent studies suggest that *v*La_max_ may peak at significantly higher cadences (> 170 rpm) than those typically used in testing protocols (120–130 rpm), highlighting the need to reassess current testing methodologies to ensure the accurate capture of true maximal values.

Although research indicates that ${\dot{\text{V}}\text{O}}_{2\text{max}}$ remains relatively stable across a range of cadences, this stability is likely confined to a specific range of movement velocities. Performance predictions made outside this range using a constant ${\dot{\text{V}}\text{O}}_{2\text{max}}$ may result in significant estimation errors.

## Conclusion

This study underscores the critical importance of considering dynamic changes in oxidative phosphorylation and glycolysis across different movement velocities when employing metabolic simulation models. Neglecting these effects can result in inaccurate intensity recommendations, potentially compromising an athlete's performance due to suboptimal training adaptations.

The analysis highlights the necessity of carefully evaluating input parameters in metabolic simulations. Incorporating cadence-dependent relationships for $\text{CE}_{{\dot{\text{V}}}\text{O2}}$ and ${{\dot {\text{V}}}{\text{O}}_{\text{2Base}}}$ may enhance the accuracy of performance predictions, especially for athletes whose performance is closely related to movement velocity. Conversely, integrating cadence-dependent changes for *v*La_max_ is not recommended. The primary challenge is to accurately determine the true maximum values, irrespective of movement velocity. This consideration is particularly important, as research in cycling indicates that *v*La_max_ may peak at substantially higher cadences than those typically utilized in performance testing. While ${\dot{\text{V}}\text{O}}_{2\text{max}}$ remains relatively stable within a specific range of movement velocities, employing a constant value outside this range may lead to significant prediction errors.

By refining the input parameters and establishing optimal protocols for their determination, the accuracy and utility of the metabolic model can be improved. This will provide more reliable predictions of endurance performance and metabolic processes, enabling researchers and practitioners to develop more effective training guidelines and pacing strategies for athletes.

## Data Availability

The datasets generated during and/or analyzed during the current study can be obtained from the corresponding author upon reasonable request.
